# Dental Implant in the Canalis Sinuosus: A Case Report and Review of the Literature

**DOI:** 10.1155/2017/4810123

**Published:** 2017-08-08

**Authors:** José Alcides Arruda, Pedro Silva, Luciano Silva, Pâmella Álvares, Leni Silva, Ricardo Zavanelli, Cleomar Rodrigues, Marleny Gerbi, Ana Paula Sobral, Marcia Silveira

**Affiliations:** ^1^Department of Oral and Maxillofacial Pathology, School of Dentistry, University of Pernambuco, Avenida General Newton Cavalcante, 1650 Aldeia dos Camarás, 54753-020 Camaragibe, PE, Brazil; ^2^Department of Oral Rehabilitation, School of Dentistry, Federal University of Goiás, Av. Primeira Avenida, s/n, Setor Leste Universitário, 74605-020 Goiânia, GO, Brazil; ^3^Faculdades Integradas da União Educacional do Planalto Central, SIGA Área Especial para Indústria, No. 2, Setor Leste, 72445-020 Gama, DF, Brazil

## Abstract

The canalis sinuosus is a neurovascular canal, a branch of nerve of infraorbital canal, through which the anterior superior alveolar nerve passes and then leans medially in course between the nasal cavity and the maxillary sinus, reaching the premaxilla in the canine and incisor region. The purpose of this article is to report a case with the presence of canalis sinuosus, in order to alert and guide professionals and discuss the morphology of this anatomical variation avoiding trans- and postsurgical disorders in dental implants. A 51-year-old female was attended to in a radiology clinic, reporting paresthesia in the right upper lip region and painful symptomatology after the installation of an implant in the corresponding region. The case revealed the presence of canalis sinuosus in imaging exams. The knowledge of this anatomical variation is essential for professionals, because attention to this region prevents irreversible damage. Therefore, the use of imaging examinations is recommended during the planning stages and treatment and after surgery in patients undergoing surgery in this area.

## 1. Introduction

The anterior superior alveolar (ASA) nerve emerges in the anterior maxillary region to innervate the incisors and canines, as well as soft tissues [1–3]. This nerve is a branch of infraorbital nerve, a branch of maxillary nerve, in the second division of the trigeminal nerve. The infraorbital nerve enters in the infraorbital canal, which has a side branch, the canalis sinuosus (CS), enabling the passage of ASA nerve [4, 5]. Canalis sinuosus is a neurovascular canal and a scarce structure, with few reports described in the literature [1, 2].

The visualization of CS, which allows passage of nervous structures to the anterior maxilla, is essential due to the frequency of CS communication with the accessory canal, also called lateral incisor canal or neurovascular variation in anterior palate [6]. In this case, the use of imaging examinations was shown to be crucial in the operative planning for installation of dental implants or other surgical procedures involving this region. Among the most used techniques are the panoramic and periapical radiographs and cone beam computed tomography (CBCT) [7].

Frequently, invasive procedures occur in the anterior maxillary region, such as dental implants, removal of supernumerary and impacted teeth, and orthognathic, endodontic, and periradicular surgeries. However, manipulation of the tissues in the anterior region may generate even irreversible damage to the patient. In particular, these losses can be neglected and iatrogenic complications may occur mainly in the region of CS location. When the patient and surgeon are made aware of the injury to the neurovascular bundle in that region, sometimes the therapeutic approach is not achieved [4, 8, 9].

The purpose of this article is to present a case with the presence of canalis sinuosus, in order to alert and guide professionals and discuss the morphology of this rare anatomical variation avoiding trans- and postsurgical disorders in dental implants in light of the literature. A Medline search from 1999 to February 2017 was conducted using the following keywords: canalis sinuosus, anatomical variation, and anatomical variation in maxilla.

## 2. Case Report

**Figure 1:**
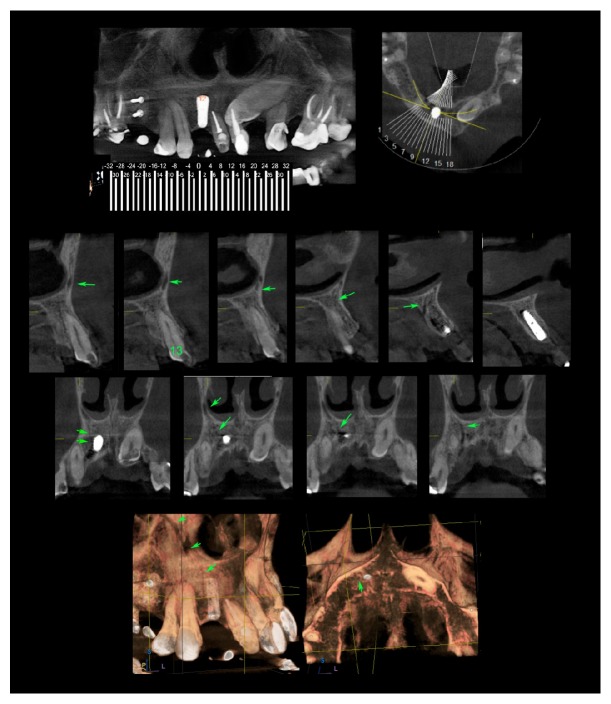
Transaxial reconstructions: hypodense course measuring about 2.0 mm wide adjacent to the top of the implant and the canine apex that extends upwards and in the anterior wall of the nasal cavity (see green arrow).

A 51-year-old female was attended to in a private radiology clinic reporting paresthesia in the right upper lip region and painful symptomatology for 22 months after the installation of an implant in the region corresponding to the right upper lateral incisor, who underwent CBCT of maxilla. No significant findings were found in extraoral and intraoral clinical examinations (preoperative imaging tests were not released by the professional). The dentist who performed the implant installation, without major intercurrences, confirmed the upper lip paresthesia reported by the patient and hypothesized two possibilities: any nerve structure lesion during surgical anesthesia and/or a psychogenic disorder. The examination was performed in tomograph cone beam (85 kVp, 7 mA, 16 bits, and FOV of 5 × 5,5 cm, in maximum resolution, in ORTHOPHOS XG 3D Ready Sirona, The Dental Company, Germany) and revealed the presence of CS, located between apical portion of the implant in lateral incisor region and the upper canine apex. The image of this canal may be observed in transaxial reconstructions as a hypodense path, measuring about 2.0 mm, adjacent to upper extreme of the implant and canine apex, extending upwards and from anterior nasal wall (Figure 1). As it is a region that involved innervation, having paresthesia as a clinical sign, the patient sought two neurologist professionals at different times. The professionals had the same opinion; that is, damage was caused in the installation of the irreversible grave implant by the time elapsed and the patient remained with a symptomatology. After expressing their opinions, the patient, together with the dentist, made a decision to no longer undergo a new surgical intervention.

## 3. Discussion

Canalis sinuosus is a neurovascular canal, nerve branch of the infraorbital canal, that passes the anterior superior alveolar nerve, initially described by Jones in 1939 and on occasion by Gray [10], being a little-known structure, with few cases described in the literature. A literature review about all CS cases was performed in the PubMed-Medline database, with only nine studies found utilizing CBCT for a better evaluation of this anatomical variation and only three case reports (Table 1).

The ASA nerve emerges in the anterior maxillary region to innervate incisors and canines, as well as soft tissues [4]. This nerve is a branch of infraorbital nerve, maxillary nerve, in the second division of the trigeminal nerve. The infraorbital nerve enters in infraorbital canal, which presents a side branch, the CS, which allows the passage of ASA nerve [2, 5].

The morphology of CS is rarely discussed in the literature and so few studies describe the length of this anatomical variation, extending about 55 mm through the maxilla, and vertical distance between the infraorbital foramen and CS may range from 0 to 9.0 mm [6]. Generally, CS presents as a bilateral structure and is, rarely, unilateral [5]. The case concerned presents a hypodense image, unilateral, measuring about 2.0 mm, adjacent to dental implant and the apex of right canine.

Manhães Júnior et al. [11] evaluated 500 CBCT examinations with intention of locating the CS and the results showed that gender did not interfere with the variations of CS. In females, mean age was 54.90 years and 53.98 years in presence or absence of CS, respectively, while in males the mean age was 56.16 years to 57.42 years when the CS was present and 55.38 years when the CS was absent. Although there were variations between the right and left sides according to distance between CS and alveolar bone crest and between CS and buccal cortical bone, the same authors showed that the location of this anatomical variation is palatine regarding superior lateral incisor. However, this study presented the need for CBCT examinations to identify anatomical variations, allowing generation of three-dimensional images, detailed assessments of these images, and treatment according to proper planning. There is no consensus between the distances of the CS. There is a variation of females and males among right and left sides regarding distances between CS and alveolar bone crest and between CS and buccal cortical bone. This may be explained by the fact that the alveolar bone plate is subject to morphological alterations over time.

Canalis sinuosus was discovered during the implantodontic treatment as described in a case of this paper. This demonstrates the necessity of dentist in knowing the existence of anatomical structure and its characteristics, which may influence the conduct of treatment and avoid complications trans- and postsurgically, as observed in first case, with presence of painful symptoms and lip paresthesia. In addition, knowledge of this region may reduce the risk of damage to neurovascular supply, as in cases of traumas in maxillofacial region, such as Le Fort I fractures, which involves the separation of maxilla with palatine region, and Le Fort II fractures, which occurs through the nasal bones and orbital rim [12].

Radiographic findings located in the periapical region are usually of odontogenic origin. However, other possibilities should be included in differential diagnosis, especially when the dental blades are preserved and pulpal sensitivity tests are positive [13]. Images of conventional periapical radiographies, panoramic radiographies, and CBCT of CS present it as being radiolucent and/or hypodense, and often the dentists are unaware of the presence of this anatomical variation. When identified, it is described as a radiolucent image in periapical region of canines and superior lateral incisors, commonly interpreted in periapical technique as an endodontic origin condition [1, 5]. Sometimes, lesions in the periapical region present a very similar radiolucent image, although the images of asymptomatic lesions in cases of malignancies, even rarely described, may show only minimal radiographic alterations, with filamentous and discreet aspect, and small changes in trabecular bone. The importance of appropriate radiograph should be highlighted, whether conventional, digital, or CBCT, and an accurate imaginologic diagnosis should be considered in order to avoid iatrogenic complications [1].

The knowledge of anatomical variations is extremely important for planning treatment and postoperatively in order to avoid complications during surgical procedures and alerting the dentist about these rare variations, ensuring a better prognosis [10]. The importance of this anatomical variation is highlighted in rehabilitation of the maxillary anterior region for the placement of implants, and the canine pillar is used as a definitive framework for support of implants, in which the contact with neurovascular bundle of CS may compromise osseointegration and cause temporary or permanent paresthesia with bleeding in situ [11].

Use of dental implants is widely used in the treatment and rehabilitation of partial or total edentulous patients. The application of imaging examinations in operative planning is essential to region analysis, as well as anatomical structures, bone quantity and quality, and presence or absence of lesions. Conventional X-ray examinations are still the alternative of most professionals who perform implantodontic technique, followed by conventional tomography and computed tomography [7].

Recently, the American Academy of Oral and Maxillofacial Radiology (AAOMR) recommended the CBCT as the best option in preoperative diagnosis for implants, providing the most suitable image for clinical evaluation [14]. However, factors such as cost and availability should also be considered [7], revealing another interface on diagnosis process of patients, confirming the need for greater attention and accuracy by the professional.

Machado et al. [15] found accessory canals of the CS by CBCT in 51.7% of 1000 patients. These data show the importance of this anatomical variation and the dentist's/surgeon's knowledge regarding the diagnosis of CS. Due to the relatively high prevalence of CS, CS identification has clinical relevance, mainly to avoid iatrogenesis in noble structures during the placement of implants in the anterior region of the maxilla. Moreover, the CBCT is one of the requests for imaging for better detection of CS.

After literature review and report of case, considering the limitations of this article, it was concluded that any surgical procedure that involves the anterior maxillary region should be evaluated regarding the presence of anatomical variation of CS, in order to prevent accidents or iatrogenic complications. And, a CBCT application is recommended to allow a possible CS identification and detail its anatomical location, diameter, length, and variation, avoiding possible iatrogenic disorders in the placement of implants or other surgical procedures involving the region.

## Figures and Tables

**Table 1:** Data of cases of the canalis sinuosus published in PubMed-Medline from 1999 to 2017.

Author, year	Gender/age	Site	Exam	Prescription	Description
Shelley et al., 1999	M/35	Upper left canine	Periapical radiography	Restorative treatment	The radiolucencies that are distinct channels with corticated borders

Neves et al., 2012	F/54	Right lateral incisor	Panoramic radiography	Implant assessment	A narrow radiolucent area, similar to a canal, adjacent to the radicular root
			CBCT		Accessory canal on the right than compared to on the left, extending from the lateral nasal wall to an accessory foramen located on the hard palate

Torres et al., 2015	F/47	Slightly medial to tooth 23	CBCT	Implant assessment	A canal extending from the lateral wall of the nasal fossa and following a course skirting its margin, up to its inferior limit

Present case 1, 2017	F/51	Between the lateral incisor region and the apex of the maxillary canine	CBCT	Implant assessment	A path adjacent to the upper end of the implant and canine apex

F: female; M: male.
